# Premenstrual syndrome and inflammatory activity in adolescent familial Mediterranean fever

**DOI:** 10.1093/rheumatology/keag169

**Published:** 2026-04-04

**Authors:** Nigar Aliyeva, Dilara Ünal, Melis Pehlivantürk Kızılkan, Özge Başaran, Yelda Bilginer, Seza Özen, Sinem Akgül

**Affiliations:** Division of Adolescent Medicine, Department of Paediatrics, Hacettepe University Faculty of Medicine, Ankara, Turkey; Department of Paediatric Rheumatology, Hacettepe University Faculty of Medicine, Ankara, Turkey; Division of Adolescent Medicine, Department of Paediatrics, Hacettepe University Faculty of Medicine, Ankara, Turkey; Department of Paediatric Rheumatology, Hacettepe University Faculty of Medicine, Ankara, Turkey; Department of Paediatric Rheumatology, Hacettepe University Faculty of Medicine, Ankara, Turkey; Department of Paediatric Rheumatology, Hacettepe University Faculty of Medicine, Ankara, Turkey; Division of Adolescent Medicine, Department of Paediatrics, Hacettepe University Faculty of Medicine, Ankara, Turkey

**Keywords:** premenstrual syndrome, familial Mediterranean fever, adolescence, inflammation, AIDAI, colchicine adherence

## Abstract

**Objectives:**

Premenstrual syndrome (PMS) comprises physical, emotional and behavioural symptoms occurring during the luteal phase of the menstrual cycle that impair quality of life. Familial Mediterranean fever (FMF) is an autoinflammatory disorder marked by chronic inflammation and recurrent attacks. This study aimed to assess the frequency and severity of PMS in adolescents with FMF, its association with disease activity, inflammatory markers and treatment adherence, and to compare findings with healthy peers.

**Methods:**

This cross-sectional study was conducted between January 2024 and February 2025. Forty adolescent girls (12–18 years) with FMF and 40 age-matched healthy controls participated. The Premenstrual Syndrome Scale (PMSS) was administered to all; in the FMF group, the Autoinflammatory Disease Activity Index (AIDAI) and colchicine adherence were also assessed. Attack frequency, menstrual features and inflammatory markers [C-reactive protein (CRP), erythrocyte sedimentation rate (ESR) and white blood cell count (WBC)] and regular colchicine use were analysed.

**Results:**

PMS frequency (*P* = 0.012) and severity (*P* = 0.032) were overall lower in the FMF group. However, PMS scores correlated positively with attack frequency and AIDAI (r = 0.368, *P* = 0.019), and ESR, CRP and WBC were higher in PMS-positive individuals. PMS-positive patients also demonstrated significantly poorer medication adherence, with higher rates of forgetting doses and discontinuing colchicine.

**Conclusion:**

PMS severity in adolescents with FMF appears linked to disease activity and inflammation. The observed relationship is consistent with an inflammatory contribution to PMS, while colchicine therapy may be associated with lower symptom burden through improved inflammation control.

## Graphical abstract

**Graphical Abstract:**
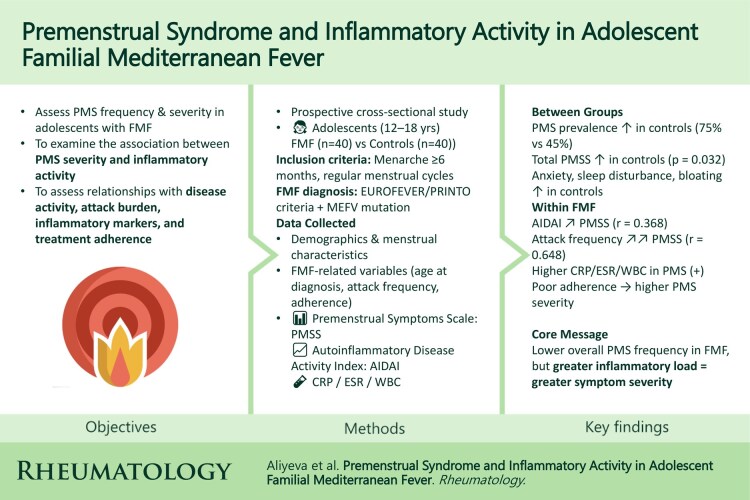

Rheumatology key messages

- Premenstrual syndrome is less frequent in adolescents with familial Mediterranean fever receiving colchicine.
- Higher disease activity and elevated inflammatory markers are associated with increased premenstrual symptom severity.
- Poor colchicine adherence may worsen premenstrual symptoms through inadequate control of underlying inflammation.

## Introduction

Premenstrual syndrome (PMS) is a common clinical condition characterized by physical, emotional and behavioural symptoms that emerge during the luteal phase of the menstrual cycle, recur periodically and can significantly impair quality of life [1]. During adolescence, PMS symptoms often begin to emerge as ovulatory cycles become established [2]. Although hormonal fluctuations are central, increasing evidence suggests that inflammatory and immunological mechanisms contribute importantly to symptom expression [3, 4].

Familial Mediterranean fever (FMF) is an autoinflammatory disease associated with mutations in the *MEFV* gene and is characterized by recurrent febrile attacks, serositis and arthritis [5–7]. One of the features of FMF is the presence of persistent subclinical inflammation, even in the absence of attacks [5–8]. The mainstay of FMF treatment is lifelong colchicine therapy, which not only reduces attack frequency and prevents complications such as amyloidosis, but also suppresses persistent subclinical inflammation [9]. Because FMF provides a human model of chronically heightened inflammatory tone, it offers a unique opportunity to examine how inflammatory burden might influence hormonally mediated symptom complexes such as PMS.

Emerging data indicate that cytokine fluctuations across the menstrual cycle may modulate mood, pain perception and somatic symptoms [10–12]. In individuals with FMF, inadequate suppression of inflammation, whether due to higher disease activity or poor treatment adherence, could therefore plausibly amplify premenstrual symptom severity [13–15]. Furthermore, recent experimental work suggests that steroid catabolites may directly activate the pyrin inflammasome, providing an additional mechanistic link between reproductive physiology and autoinflammatory pathways [16].

Despite this biologic rationale, clinical data examining PMS in adolescents with autoinflammatory diseases are scarce. Clarifying this relationship may improve symptom recognition, support adherence strategies and enhance multidisciplinary care.

Therefore, we aimed to compare the frequency and severity of PMS in adolescents with FMF and healthy peers, and to evaluate whether premenstrual symptoms vary according to inflammatory activity, attack burden and colchicine adherence.

## Methods

### Study design and ethical approval

This cross-sectional and analytical study was conducted between June 2024 and February 2025 at the Divisions of Adolescent Medicine and Pediatric Rheumatology, Hacettepe University İhsan Doğramacı Children’s Hospital. The study included 40 adolescent girls aged 12–18 years diagnosed with FMF and 40 age-matched healthy controls who voluntarily participated.

Written informed consent was obtained from all participants and their legal guardians. Data collection was carried out in accordance with confidentiality and ethical principles. The study was approved by the Non-Interventional Clinical Research Ethics Committee at Hacettepe University (Approval No: SBA 24/908).

### Inclusion and exclusion criteria

The inclusion criteria for both the FMF group and the healthy control group required participants to be between 12 and 18 years of age, to have experienced menarche at least six months prior, and to have a regular menstrual cycle. Regular menstrual cycle was defined, in accordance with the American Academy of Pediatrics (AAP) and the American College of Obstetricians and Gynecologists (ACOG) guidelines, as a cycle length between 21 and 45 days with a duration of bleeding ≤7 days [17].

For participants in the FMF group, an additional requirement was a confirmed diagnosis of FMF according to the EUROFEVER/PRINTO criteria and the presence of either a homozygous or compound heterozygous mutation in exon 10 of the *MEFV* gene.

Exclusion criteria common to both groups included unwillingness of the participant or their family to take part in the study, the presence of an additional chronic disease, taking any medication that could affect the menstrual cycle or mood, intellectual disability or a psychiatric diagnosis that would prevent the completion of the study questionnaires.

### Data collection instruments and measurements

Information on participants’ age, age at menarche, cycle length, cycle regularity, duration and amount of menstrual bleeding, and the presence of dysmenorrhea was obtained through face-to-face interviews. Menstrual bleeding lasting 7 days or longer was classified as ‘prolonged’, while the use of six or more pads per day was classified as ‘heavy menstrual bleeding’. Dysmenorrhea was recorded as present or absent based on participants’ self-report.

Height and weight measurements of all participants were used to calculate body mass index (BMI) in kilograms per square meter (kg/m^2^). In the FMF group, systolic and diastolic blood pressure values were evaluated according to age-, sex- and height-specific percentile tables. Blood pressure values above the 95th percentile were considered hypertension, while values below the fifth percentile were considered hypotension [18]. Systemic physical examination was conducted and pubertal staging was assessed using the Tanner–Marshall method.

For participants with FMF, age at diagnosis, type of genetic mutation, number and type of attacks in the past 6 months, medication use and laboratory findings [C-reactive protein (CRP), erythrocyte sedimentation rate (ESR), haemoglobin (Hb), white blood cell count (WBC), platelet count (PLT)] were obtained from patient files and the electronic medical record system. If an FMF attack had occurred within the preceding six months, laboratory measurements obtained during that episode were used. In the absence of an attack, values from the most recent routine follow-up visit within the same period were recorded. Information regarding menstrual phase at the time of sampling was not available.

Patients were classified according to the number of attacks in the past six months as having no attacks (0 attacks), one attack, two attacks or three or more attacks (≥3 attacks). For analytical purposes, participants were also grouped as having a single attack or multiple attacks. All participants completed the Premenstrual Symptoms Scale (PMSS), while only the FMF group additionally completed the Autoinflammatory Disease Activity Index (AIDAI) to evaluate disease activity. Medication adherence was evaluated using the following three questions: Do you ever forget to take your medication? Do you always take your medication on time? Have you ever discontinued your medication?

### Premenstrual syndrome scale (PMSS)

Premenstrual symptoms were assessed using the validated 44-item Premenstrual Syndrome Scale (PMSS), a five-point Likert instrument that provides a total score ranging from 44 to 220. The PMSS consists of nine subscales: depression, anxiety, irritability, fatigue, concentration difficulties, pain, appetite, sleep and bloating. In accordance with established thresholds, scores ≥110 were considered indicative of PMS [19]. Participants were therefore categorized as PMS-positive or negative based on this cut-off. In addition, scores between 111 and 148 reflect mild PMS, 149 and 184 indicate moderate PMS and 185 and 220 represent severe PMS. The original version of the scale demonstrated a Cronbach’s alpha (α) of 0.75, while in the present study α was calculated as 0.95.

### Autoinflammatory disease activity index (AIDAI)

Disease activity was assessed using the validated Autoinflammatory Disease Activity Index (AIDAI), which records the presence of 12 FMF-related symptoms over the preceding 30 days and yields a total score between 0 and 12. These symptoms are fever (≥38°C), general attack manifestations, abdominal pain, nausea/vomiting, diarrhoea, headache, chest pain, painful lymph nodes, arthralgia or myalgia, joint swelling, ocular findings and skin rash. Each symptom is scored by the patient as 0 (absent) or 1 (present). The total score reflects the level of disease activity: 0–3 indicates mild, 4–6 moderate and 7–12 severe disease activity. Higher scores indicate active disease status, with a reported Cronbach’s alpha reliability coefficient of >0.80 [20]. For the purposes of this study, AIDAI was dichotomized as 0 *vs* >0 to distinguish complete remission from any detectable inflammatory activity, thereby enabling a sensitive evaluation of inflammatory burden within the cohort.

### Data analysis and statistical analysis

The research data were analysed using SPSS version 25.0 (IBM Corp., Armonk, NY, USA). The normality of numerical variables was assessed using the Kolmogorov–Smirnov test. Continuous variables with normal distribution were presented as mean ± standard deviation, while those without normal distribution were expressed as median (minimum–maximum). For comparisons between two groups, the Independent Samples *t* test was used for normally distributed continuous variables, and the Mann–Whitney *U* test was applied for non-normally distributed variables. For comparisons among three or more groups, one-way analysis of variance (ANOVA) was used for normally distributed variables, and the Kruskal–Wallis test was performed for non-normally distributed variables, followed by appropriate post-hoc pairwise comparisons when applicable. For categorical variables, the Pearson χ^2^ test or Fisher’s exact test, when appropriate, was employed. Correlation analyses between continuous variables were performed using Pearson’s correlation coefficient (r) and interpreted as weak (r = 0.00–0.29), moderate (r = 0.30–0.69) or strong (r = 0.70–1.00). A *P*-value of <0.05 was considered statistically significant for all analyses.

## Results

A total of 60 adolescents with FMF and 48 healthy controls were screened for eligibility. In the control group, eight individuals were excluded (five due to menstrual irregularity and three because they declined to complete the questionnaires). In the FMF group, 20 patients were excluded (five menstrual irregularity, two refusal to complete questionnaires and 13 not meeting the predefined genetic criteria). Consequently, 40 participants with FMF and 40 age-matched controls were included in the final analysis. Within the FMF cohort, 18 participants (45%) were classified as PMS-positive and 22 (55%) as PMS negative. In the control group, PMS was present in 30 participants (75%) and absent in 10 (25%).

### Anthropometric and clinical data

The mean age of the FMF group was 16.2 ± 1.3 years and that of the control group was 16.1 ± 1.5 years (*P* = 0.623). Mean height was significantly lower in the FMF group compared with controls (160.5 ± 5.7 cm *vs* 163.5 ± 4.9 cm, *P* = 0.010), while mean weight (52.7 ± 5.6 kg *vs* 55.7 ± 12.4 kg, *P* = 0.144) and BMI (20.1 ± 1.53 *vs* 20.8 ± 1.43 kg/m^2^, *P* = 0.130) did not differ significantly.

### Disease characteristics in FMF group

Among the participants, 52.5% carried homozygous mutations and 47.5% had compound heterozygous (pathogenic) mutations. In the preceding six months, 55% reported no attacks, while most of the remainder experienced only one to two attacks. When present, attacks frequently involved multiple symptoms, most commonly abdominal pain, fever and joint pain.

### Evaluation of menstrual data

The mean age at menarche was similar between the FMF group and controls (12.5 ± 1.0 *vs* 12.3 ± 1.1 years, *P* = 0.075). The prevalence of dysmenorrhea did not differ significantly (52.5% *vs* 42.5%, *P* = 0.370). However, menstrual characteristics showed notable differences between the groups: prolonged menstruation (≥7 days) was more frequent in the FMF group compared with controls (40.0% *vs* 17.5%, *P* = 0.026) and heavy menstrual bleeding (≥6 pads/day) was also significantly higher in the FMF group (35.0% *vs* 15.0%, *P* = 0.039). PMS was observed in 45% of the FMF group, whereas 55% of the patients did not exhibit any PMS-related symptoms (Table 1).

**Table 1:** Descriptive clinical characteristics of the FMF group.

Variable	Subgroup	*n* (%)
Homo/Heterozygous	Homozygous	21 (52.5)
	Compound heterozygous	19 (47.5)
Attack frequency (in the last 6 months)	No attacks	22 (55.0)
	1 attack	8 (20.0)
	2 attacks	9 (22.5)
	≥3 attacks	1 (2.5)
Type of attack Type of Attack	Single symptom	11 (27.5)
	Multiple symptoms	29 (72.5)
Group assignment	PMS +	18 (45.0)
	PMS −	22 (55.0)
Colchicine use	40 (100.0)	
Colchicine non-use	0 (0.0)	

Data are presented as *n* (%). FMF: familial Mediterranean fever; PMS: premenstrual syndrome.

### PMS data: comparison of frequency and severity between groups

As shown in Table 2, the overall PMSS score was significantly higher in the control group than in the FMF group (*P* = 0.032). Moreover, PMS was more frequently observed in controls compared with patients with FMF (*P* = 0.012). Subscale scores for anxiety, sleep disturbance and bloating were also found to be more elevated in the control group (*P* = 0.001, *P* < 0.001 and *P* < 0.001, respectively).

**Table 2:** Comparison of PMS scale scores and symptom subdimensions between the FMF and control groups.

Variable	FMF group (*n* = 40)	Control group (*n* = 40)	*P*-value
PMS scale score	112.9 ± 28.4	126.3 ± 26.6	**0.032**
Presence of PMS, *n* (%)	18 (45)	30 (75.0)	**0.012**
PMS severity, *n* (%)			
No PMS, *n* (%)	22 (55.0)	10 (25)	**0.003**
Mild PMS (110–150), *n* (%)	15 (37.5)	23 (57.5)	0.117
Severe PMS (> 150), *n* (%)	3 (7.5)	7 (17.5)	0.310
Subscales			
Depression	29.1 ± 11.2	25.9 ± 6.6	0.121
Anxiety	12.5 (8–28)	19.0 (8–33)	**0.001**
Irritability	15.5 (7–23)	20.0 (5–25)	0.199
Attention	12.0 (3–15)	11.5 (4–20)	0.325
Pain	9.0 (3–15)	8.0 (3–15)	0.375
Appetite	9.0 (3–15)	8.0 (3–15)	0.698
Sleep	7.7 ± 2.7	12.1 ± 4.0	**0.001**
Bloating	3.0 (3–15)	9.0 (3–19)	**0.001**
Fatigue	12.8 ± 3.8	11.4 ± 4.1	0.118

Data are presented as mean ± standard deviation or median (minimum–maximum), as appropriate, and as *n* (%). Comparisons between the FMF and control groups were performed using the independent samples *t* test for normally distributed continuous variables and the Mann–Whitney *U* test for non-normally distributed variables. Categorical variables were compared using the χ^2^ test or Fisher’s exact test. Bold text highlights significant results. FMF: familial Mediterranean fever; PMS: premenstrual syndrome.

### Clinical and laboratory parameters of the FMF group according to PMS status

In the FMF group, PMS-positive individuals had significantly higher AIDAI scores (*P* = 0.017) and attack frequency (*P* < 0.001) compared with PMS-negative individuals. Laboratory parameters also differed, with ESR (*P* = 0.029), CRP (*P* = 0.039) and WBC counts (*P* = 0.004) being significantly elevated in the PMS-positive group. No significant differences were observed for genotype distribution, platelet count, haemoglobin levels or type of attack (Table 3).

**Table 3:** Comparison of inflammatory and haematological markers between PMS-positive and PMS-negative patients with FMF.

Parameter	Subgroup	PMS (+) (n = 18)	PMS (−) (*n* = 22)	*P*-value
Genetic, N (%)	Homozygous	12 (%66.7)	9 (%40.9)	0.125
	Heterozygous	6 (%33.3)	13 (%59.1)	
AIDAI, Median (IQR)		2 (0–5)	0 (0–4)	**0.017**
Attack frequency in the past 6 months, n (%)	Median (IQR)	1.42 (0–3)	0.1 (0–1)	**<0.001**
	0	3 (%16.7)	19 (%86.4)	
	1	6 (%33.3)	2 (%9.1)	
	2	8 (%44.4)	1 (%4.5)	
	≥3	1 (%5.6)	0 (%0.0)	
Type of Attack, *n* (%)	**Single symptom**			
	Fever	2 (%11.1)	0 (%0.0)	0.219
	Abdominal pain	2 (11.1%)	4 (%18.2)	0.664
	Arthralgia	2 (%11.1)	1 (%4.5)	0.596
	**Multiple symptoms**	13 (%72.2)	16 (%72.7)	>0.999
ESR (mm/h), Median (IQR)		19.0 (2–60)	12.0 (2–26)	**0.029**
CRP (mg/L), Median (IQR)		6.0 (0.4–143)	3.1 (0.3–17.4)	**0.039**
WBC (10³/µL), Mean ± SD		8.2 (5.3–17.0)	6.4 (3.2–15.7)	**0.004**
PLT (10³/µL), Mean ± SD		293.5 ± 78.0	329.8 ± 71.9	0.134
Hb (g/dL), Mean ± SD		12.5 ± 1.5	12.6 ± 1.5	0.895

Bold text highlights significant results. AIDAI: Autoinflammatory Disease Activity Index; CRP: C-reactive protein; ESR: erythrocyte sedimentation rate; FMF: familial Mediterranean fever; Hb: haemoglobin; PLT: platelet count; PMS: premenstrual syndrome; WBC: white blood cell count.

In the FMF group, the AIDAI score was analysed in relation to PMS outcomes. Patients with an AIDAI score of 0 had significantly lower PMSS scores than the control group, whereas no significant difference was observed between patients with AIDAI >0 and controls (Table 4). A moderate positive correlation was found between AIDAI and PMSS scores (*P* = 0.019, r = 0.368).

**Table 4:** Comparison of PMS characteristics among controls and patients with FMF with low and high disease severity.

		FMF		Control (c) *n* = 40	*P*-value	FMF		Control (c) *n* = 40	*P*-value	FMF		Control (c) *n* = 40	*P*-value
						No attacks (a) *n* = 21	≥1 attack (b) *n* = 19			Homozygous (a) *n* = 21	Heterozygous (b) *n* = 19		
		AİDAİ = 0 (a) *n* = 20	AİDAİ > 0 (b) *n* = 20										
**PMS scale Total score,** Median (IQR)		96 (84.7–105.7)	129.5 (110.2–142)	133.5 (110.2–142)	**0.004** **a < b = c**	94.4	132.7	126.4	**<0.001** **(a/b)** **<0.001** **(a/c)** 1.000 (b/c)	116.5	108.8	126.4	0.347 (a/b) 0.271 (a/c) **0.015 (b/c)**
**Presence of PMS,** *n* (%)		5 (25)	15 (75)	30 (75.0)	**0.001** **a < b = c**	2 (4.2)	16 (33.3)	30 (62.5.0)	**<0.001** **(a/b)** **<0.001** **(a/c)** 1.000 (b/c)	12 (57.1)	6 (31.6)	30 (75.0)	0.244/**0.004**
**PMS severity,** *n* (%)	Mild (110–150)	3 (15)	12 (60)	23 (57.5)	**0.001** **a < b = c**	2 (5.3)	13 (34.2)	23 (60.5)	**<0.001** **a < b = c**	10 (47.6)	5 (26.3)	23 (57.5)	0.590/**0.030**
	Severe (>150)	1 (5)	2 (10)	7 (17.5)		0 (0.0)	3 (30)	7 (70)		2 (9.5)	1 (5.3)	7 (17.5)	0.479/0.416

For comparisons involving three or more groups, one-way ANOVA or the Kruskal–Wallis test was applied according to data distribution. Bold text highlights significant results.

FMF: familial Mediterranean fever; PMS: premenstrual syndrome; AIDAI: Autoinflammatory Disease Activity Index.

When patients with FMF were stratified by attack frequency, PMSS scores were significantly lower in the subgroup without attacks compared with both the control group and the subgroup with ≥1 attacks. However, no significant difference was observed between the subgroup with ≥1 attacks and the control group. Moreover, PMS presence and severity were significantly lower in the no-attack subgroup compared with controls (Table 4). A strong positive correlation was also identified between attack frequency and PMSS scores (*P* < 0.001, r = 0.648).

When participants were compared by genetic mutation status, no significant differences in PMSS scores were observed between the homozygous subgroup and either the heterozygous or control groups. However, the heterozygous subgroup differed significantly from controls, with lower PMSS scores (*P* = 0.015), reduced PMS prevalence (*P* = 0.004) and lower rates of mild PMS severity (*P* = 0.030) (Table 4).

Medication adherence behaviours were compared between PMS-positive (*n* = 18) and PMS-negative (*n* = 22) patients with FMF (Table 5). PMS-positive patients were significantly more likely to report forgetting to take their medication compared with PMS-negative patients (88.9% *vs* 40.9%, *P* = 0.005). In addition, a markedly higher proportion of PMS-positive patients reported having discontinued their medication at least once (88.9% *vs* 9.1%, *P* < 0.001), indicating substantially poorer adherence in this group. Although a lower proportion of PMS-positive patients reported taking their medication on time compared with PMS-negative patients (55.6% *vs* 72.7%), this difference did not reach statistical significance (*P* = 0.424).

**Table 5:** Comparison of medication adherence behaviours between PMS-positive and PMS-negative patients with FMF.

Variable, *n*(%)		PMS (+) (*n* = 18)	PMS (−) (*n* = 22)	*P*
Do you ever forget to take your medication?	Yes	16 (%88.9)	9 (%40.9)	**0.005**
	No	2 (%11.1)	13 (%59.1)	
Do you always take your medication on time?	Yes	10 (%55.6)	16 (%72.7)	0.424
	No	8 (%44.4)	6 (%27.3)	
Have you ever discontinued your medication?	Yes	16 (%88.9)	2 (%9.1)	**<0.001**
	No	2 (%11.1)	20 (%90.9)	

Data are presented as *n* (%). Comparisons between PMS-positive and PMS-negative patients with FMF were performed using the χ^2^ test or Fisher’s exact test. Bold text highlights significant results. PMS: premenstrual syndrome; FMF: familial Mediterranean fever.

## Discussion

Although the impact of systemic inflammation on premenstrual symptoms has long been debated, this relationship has not been fully elucidated in the context of autoinflammatory diseases. In this regard, the present study aimed to evaluate the frequency and severity of PMS in adolescents diagnosed with FMF and to analyse its association with disease activity, inflammatory markers and treatment adherence. In this cohort, we observed a paradoxical pattern: overall PMS frequency was lower than in healthy peers, yet symptom severity increased in parallel with higher inflammatory activity. Disease activity indices, attack burden and acute-phase markers all showed coherent associations with PMSS scores. Together, these findings support the concept that inflammatory load modulates premenstrual symptom expression.

Furthermore, the association between regular colchicine adherence and milder PMS symptoms is consistent with the possibility that better inflammation control may be linked to reduced hormonal symptom burden.

In fact, treatment non-adherence behaviours such as forgetting to take medication and voluntarily discontinuing therapy were found to be more common among PMS-positive patients with FMF compared with PMS-negative ones. Non-adherence may lead to insufficient suppression of inflammation, resulting in increased disease activity and, consequently, the exacerbation of both physical and psychological PMS symptoms. Similar findings have also been reported in the literature. In a study conducted among paediatric patients, those with poor adherence to treatment had significantly higher depression scores [13]. In contrast, another study involving adults aged 19–64 years demonstrated that individuals who regularly used their medication had lower levels of anxiety, which was possibly associated with better control of inflammation [14]. Likewise, Duruoz et al. reported that fatigue in patients with FMF was associated with disease duration, attack frequency and treatment adherence [15]. These observations suggest that inflammation in FMF is not merely a biological process but also part of a psychoneuroimmunological response influenced by treatment adherence, which may modulate the severity of PMS symptoms.

The observation that menstrual bleeding abnormalities were more common in the FMF group despite lower PMS burden may appear paradoxical. One explanation relates to continuous anti-inflammatory therapy: effective suppression of inflammatory activity with colchicine may be associated with reduced expression of premenstrual symptoms. Because all patients with FMF were receiving colchicine, their overall inflammatory milieu may have differed from that of controls, who were not exposed to such treatment.

However, pharmacologic modulation is unlikely to be the sole factor. Adolescents living with a chronic inflammatory disease may develop adaptive coping strategies or altered symptom appraisal, potentially influencing how cyclical emotional or somatic changes are perceived and reported. Regular medical follow-up and greater health awareness may further promote earlier recognition and improved self-management of symptoms [21]. In addition, variations in expectation, reporting behaviour or thresholds for distress cannot be excluded. Together, these mechanisms may contribute to the divergence between menstrual bleeding characteristics and PMS severity.

Previous studies have reported that hormonal fluctuations can enhance inflammatory responses and exacerbate PMS symptoms. In particular, the decline in oestrogen levels, accompanied by an increase in IL-6, has been shown to trigger inflammation during menstruation and may influence the inflammatory process in FMF. Ben-Chetrit *et al.* reported menstruation-related attack exacerbations in 7% of women with FMF [22], while Karadağ *et al.* observed such increases in 33.7% of patients, supporting the notion that the menstrual cycle can modulate inflammatory activity [23]. Our findings suggest that colchicine therapy may attenuate these inflammatory fluctuations, thereby alleviating PMS symptoms. This indicates that not only the presence but also the intensity and control of inflammation through treatment may play a decisive role in the development and persistence of PMS symptoms. Consistent with this notion, studies in other rheumatologic diseases such as rheumatoid arthritis and systemic lupus erythematosus have shown that women with higher disease activity exhibit a significantly increased prevalence of PMS and premenstrual dysphoric disorder (PMDD) [24, 25].

In the FMF group, increase in attack frequency and AIDAI scores were accompanied by higher PMSS scores; a moderate-to-strong positive correlation was observed between attack frequency and PMS severity, while a significant positive correlation was identified between AIDAI and PMSS scores. These findings suggest that the chronic inflammatory nature of FMF may play a role in the manifestation of PMS symptoms. Similar clinical evidence supporting the influence of inflammation on PMS symptoms has been reported in the literature. Yama *et al.* (2020) demonstrated significantly elevated IL-10 levels during the luteal phase in women with severe PMS symptoms [26], whereas Azizieh *et al.* found positive correlations between IL-10 and IL-12 levels and PMS symptom severity [27]. Collectively, these findings indicate that systemic inflammation may interact with sex hormones to exacerbate neuropsychiatric manifestations and that pro-inflammatory cytokines could influence the neuroendocrine system, thereby increasing the severity of PMS symptoms. Since sex hormones are steroid hormones, this effect may also be explained by a recent study of Magnotti *et al.* These authors have shown that endogenous steroid catabolites may serve as unconventional pyrin activation mechanisms, thus enhancing inflammation [16].

When inflammatory markers were evaluated, ESR, CRP and WBC levels were found to be significantly higher in PMS-positive individuals with FMF. This finding supports the potential role of inflammation in the manifestation of PMS symptoms. Similarly, Anirudh *et al.* reported higher ESR, WBC and CRP levels in individuals diagnosed with PMS compared with controls, although these markers were not directly associated with symptom severity. Moreover, women with CRP levels above 3 mg/l were shown to experience more pronounced physical and emotional symptoms during the premenstrual period [28]. Gaskins *et al.* also demonstrated that CRP levels fluctuate throughout the menstrual cycle, reaching their peak during menstruation [29]. These observations suggest that the relationship between PMS and inflammation is influenced by individual variability and cyclic hormonal changes.

This study has several limitations. First, the relatively small sample size limited the statistical power, particularly in subgroup analyses. The single-centre and cross-sectional design also restricts the generalizability of the findings to broader populations. PMS was assessed using a single-time self-reported measure. Prospective daily ratings across the menstrual cycle would allow more precise phase-specific evaluation; however, such approaches can be difficult to implement in adolescent cohorts. Another limitation is the inability to precisely determine whether FMF attacks coincided with the premenstrual period, as the questionnaires were designed to assess general symptom patterns rather than cycle-specific timing. Consequently, the temporal relationship between PMS symptoms and FMF activity may not have been fully captured. Furthermore, none of our patients were completely non-adherent to colchicine therapy, which made it difficult to evaluate the relationship between FMF and PMS in the absence of treatment. Designing such a study would be challenging due to ethical concerns regarding withholding colchicine, which is the standard of care for FMF. Another limitation concerns the categorization of disease activity. To preserve statistical sensitivity within a modest sample, AIDAI was dichotomized as 0 *vs* >0 rather than using validated severity thresholds. This strategy aimed to capture low-level or transient symptoms and therefore reflect general inflammatory burden rather than formally defined active disease. Finally, medication adherence was assessed using three self-reported questions rather than a validated adherence scale, which may limit the precision of adherence measurement Considering these limitations, future research with larger sample sizes, prospective design and a specific focus on attack periods is warranted to clarify the relationship between PMS and FMF more accurately.

## Conclusion

While overall PMS frequency was lower in adolescents with FMF receiving colchicine, symptom severity rose in parallel with increasing disease activity and inflammatory burden. Premenstrual worsening may therefore represent a clinically relevant signal of suboptimal inflammation control and potential non-adherence. Incorporating menstrual symptom assessment into routine care could provide an additional, patient-centred tool to support disease monitoring and interdisciplinary management.

## Data Availability

The data supporting the findings of this study were generated from clinical assessments and questionnaires administered to adolescent participants. Due to ethical considerations and the protection of participant confidentiality, the data are not publicly available. However, anonymized data may be made available from the corresponding author upon reasonable request and with appropriate ethical approval.
